# Extramedullary Multiple Myeloma: Challenges and Opportunities

**DOI:** 10.3390/curroncol32030182

**Published:** 2025-03-20

**Authors:** Matthew Ho, Luca Paruzzo, Janna Minehart, Neel Nabar, Julia Han Noll, Thomas Luo, Alfred Garfall, Saurabh Zanwar

**Affiliations:** 1 Division of Hematology, University of Pennsylvania, Philadelphia, PA 19104, USA; homat@pennmedicine.upenn.edu (M.H.); luca.paruzzo@pennmedicine.upenn.edu (L.P.); janna.minehart@pennmedicine.upenn.edu (J.M.); neel.nabar@pennmedicine.upenn.edu (N.N.); jhnoll@pennmedicine.upenn.edu (J.H.N.); thomas.luo@pennmedicine.upenn.edu (T.L.); alfred.garfall@pennmedicine.upenn.edu (A.G.); 2 Division of Hematology, Mayo Clinic, Rochester, MN 55905, USA

**Keywords:** relapsed myeloma, paraskeletal disease, MAPK, CAR-T, bispecific antibodies

## Abstract

Extramedullary multiple myeloma (EMM), defined in this review as soft tissue plasmacytomas resulting from hematogenous spread, is characterized by the ability of MM cells to proliferate outside of the bone marrow microenvironment. It is aggressive, often associated with high-risk cytogenetics and early relapse, and independently portends significantly shorter progression-free and overall survival, even in the era of highly effective immunotherapies. The molecular and microenvironmental factors underlying extramedullary MM dissemination continue to be studied to inform the development of better treatments. In this review, we discuss our current understanding of the biology of EMM, focusing on its distinct molecular and microenvironmental characteristics vis-à-vis MM. We also review the current treatment strategies, acknowledging the paucity of large, randomized studies specific to this population.

## 1. Introduction

Multiple myeloma (MM) is a cancer of plasma cells arising within the bone marrow (BM), and is dependent on the marrow microenvironment for survival and proliferation [1]. The MM cell originates from a post-germinal center B cell that has undergone sequential rounds of hypermutation and antigenic selection through the class-switch recombination process in lymph node germinal center [2]. However, this is not sufficient to result in MM, as the MM clone needs to home into the BM for survival. There, over time, it can induce immune microenvironment changes and form specialized micro-niches, which underlie the patchy distribution of myeloma tumor cells within the BM [3]. Within this favorable sanctuary, MM cells acquire secondary genetic aberrations and accumulate a sufficient clonal burden to eventually progress to symptomatic MM [1]. The majority of the classical myeloma clinical features are the direct consequence of disrupted homeostasis within the BM microenvironment—anemia from MM replacement and suppression of normal BM progenitor cells, lytic bone lesions, and hypercalcemia from altered bone metabolism [4].

Less commonly, clonal plasma cells can form tumors at anatomical sites outside of the BM: (1) extramedullary MM (EMM), defined as MM with visceral and soft tissue disease not contiguous with the bone, and (2): MM with paraskeletal disease (PSD), defined as MM with plasmacytomas growing contiguously from BM lesions following cortical bone disruption. Importantly, solitary plasmacytoma—defined as a single extramedullary cluster of plasma cells with <10% clonal plasma cells in the BM—does not meet the diagnostic criteria for MM and is not considered EMM or PSD [5]. Additionally, plasma cell leukemia (PCL), defined by the International Myeloma Working Group (IMWG) as the presence of ≥5% circulating plasma cells in the peripheral blood [6], is a distinct entity that is commonly excluded from contemporary definitions of EMM.

There is currently no standard definition of EMM, and historical definitions have included varying permutations of these presentations [7,8,9,10,11,12,13]. An unambiguous definition of EMM is important, especially because certain patterns of extramedullary involvement have emerged as poor prognostic markers independent of the revised international staging system (R-ISS) stage, high-risk cytogenetics, or type of therapy [14]. The emerging consensus on the definition of EMM have excluded paraskeletal disease, which appears to have better outcomes than EMMbased on various retrospective analyses (Table 1) [15]. In a uniformly treated cohort of 351 patients with RRMM receiving ide-cel, a BCMA-directed CAR-T therapy, patients with EMM had a significantly inferior PFS (hazard ratio of 1.7) compared to PSD [16]. Similarly, in an EBMT study of patients undergoing autologous stem cell transplant, EMM had significantly inferior outcomes compared to PSD with a 3-year overall survival of 58% for EMM versus 78% for PSD (*p* < 0.001) [17]. In line with this, we define EMM as an extraosseous clonal plasma cell mass involving soft tissue or organs not contiguous with bone lesions.

## 2. Epidemiology

The true incidence of EMM is hard to accurately ascertain and varies considerably owing to its rarity, differing definitions in the past, as well as heterogeneity in imaging modalities utilized for the assessment of bony lesions and inconsistent surveillance in the absence of suspicious symptoms or other clinical finding. Nonetheless, the overall incidence of EMM has been reported to range from 0.5 to 5.2% at diagnosis (primary EMM) and 5-30% at relapse (secondary EMM); PSD seems to be more common, present in 7–32% of patients at diagnosis and around 10-30% at relapse for secondary paraskeletal plasmacytoma (Table 1) [10,11,17,19,20,21,22,23,24,26,27,28,29,30,31,32,33].

EMM can involve virtually any organ, including the central nervous system (CNS) [34], lymph nodes [35], spleen [35], breast [36], thyroid [36], throat [37], lungs [38], gastrointestinal tract [39], liver [35], heart [40], pancreas [41], reproductive organs [42,43], skin [44], muscle [45], and adrenal glands [46]. A retrospective review of MM patients treated at the Mayo Clinic between Jan 2000 and Dec 2021 identified 299 pathology-proven patients with EMM, of which 157 (52%) had visceral disease and 142 (48%) had non-visceral disease [47]. The most common visceral sites included liver (32%) followed by lung/pleural effusion (31%). The most common non-visceral sites are soft tissue/musculoskeletal (excluding PSD) (78%) followed by lymphadenopathy (30%) [47]. While there was a trend toward inferior survival for visceral EMM, this was not noted to be independently prognostic [47].

## 3. Pathogenesis of Multiple Myeloma Within the Context of the BM Milieu

Both EMM and MM are characterized by the clonal expansion of malignant plasma cells that have undergone somatic hypermutation and class-switch recombination. Class-switch recombination, specifically, is error-prone and can result in chromosomal translocations between the immunoglobulin heavy-chain (IgH) locus (which contains strong enhancers) and oncogenes such as cyclin D1/t (11;14), cyclin D3/t (6;14), FGFR3/t (4;14), MAF/t (14;16), and MAFB/t (14;20) [48]. These primary IgH translocations account for roughly half of the initiating events, and gain of odd-numbered chromosomes (hyperdiploidy) accounts for the rest [49]. Analogous to a long-lived plasma cell, the transformed clone then needs to establish a niche within the BM to survive, giving rise to monoclonal gammopathy of undetermined significance (MGUS), a premalignant condition characterized by a low tumor burden and the potential to accumulate a sufficient tumor burden to cause symptomatic MM [50]. Supporting this, epidemiological studies have shown that MGUS consistently precedes MM (i.e., all MM evolves from MGUS) and progresses to MM at a rate of about 1% per year (i.e., not all MGUS becomes MM) [51,52].

Several key pathways facilitate MM marrow infiltration, and chief among them is the CXCL12-CXCR4 axis. Myeloma cells highly express CXCR4 [53], a chemokine receptor that interacts with CXCL12, which in turn is highly expressed in the BM microenvironment and functions canonically to support quiescence and BM retention of hematopoietic stem cells (HSCs) [54]. Consistent with this, the CXCR4 inhibitor plerixafor disrupts MM-BM interaction [55] and rapidly mobilizes MM in the peripheral blood [56]. Importantly, a phase I/II trial assessing weekly plerixafor in relapsed/refractory MM (RRMM) did not report any extramedullary disease or plasma cell leukemia at the median follow-up of 5.5 (range 0.5–34) months [56], suggesting that extramedullary dissemination of MM relies on more than just its ability to leave the BM. Other important chemokines that mediate MM trafficking into the BM include MIP-1α/CCL3 (receptors: CCR1, CCR3), MIP-1β (receptor: CCR5), RANTES/CCL5 (receptors: CCR1, CCR3, CCR5), and IL-8/CXCL8 (receptor: CXCR1, CXCR2) [57]. CXCL12-CXCR4 engagement also upregulates adhesion molecules to promote firm adhesion to the fenestrated BM sinusoidal endothelium, a crucial step for MM transmigration through the endothelial layer, basal lamina, and into the BM [58]. The key adhesion molecules expressed by MM include α4β1 integrin (binds VCAM-1), α4β7 integrin (binds MAdCAM-1 and fibronectin), CD44 (binds hyaluronic acid), PSGL-1 (binds P- and E-selectin), and CD56 [58]. Within the BM, α4β1, α4β7, and CD56 function to anchor MM cells, preventing egress [59,60,61,62].

Direct contact and paracrine interactions between MM and the various BM compartments (stromal, endothelial, immune, and osteogenic) reprogram the BM niche [63,64]. For example, BM stromal–MM cell adhesion upregulates NFkB-dependent transcription and IL-6 secretion, as well as other prosurvival pathways such as MAPK/ERK, JAK/STAT, and Pi3K/Akt [65]. This creates a permissive environment that the myeloma-initiating clone is dependent on for survival, where it can acquire secondary genetic aberrations (e.g., del(17p13), gain(1q), and del(1p)) [66] and accumulate a sufficient clonal burden to cause symptomatic MM.

## 4. Pathophysiology of Extramedullary Multiple Myeloma

Current evidence supports the notion that extramedullary spread occurs after multiple myeloma (MM) has homed to the BM and eventually loses BM dependency and anchorage, allowing EMM to egress, survive, and proliferate outside the BM [67,68].

### 4.1. Insights from Immunophenotypic Studies

**Figure 1:**
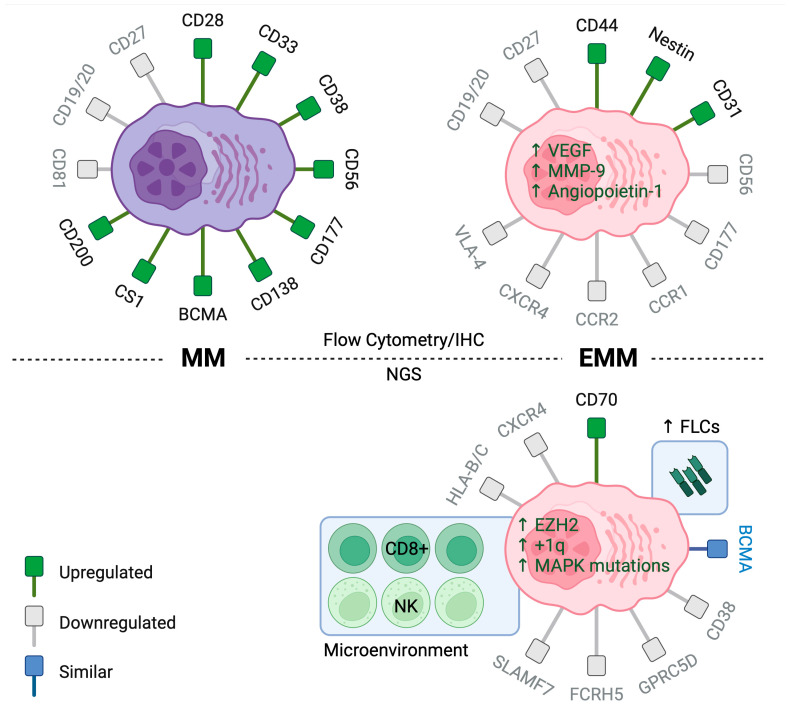
Immunophenotype of MM (**Left**) and EMM (**Right**). **Top**: Flow cytometry and immunohistochemistry analyses reveal significant differences in the expression of chemotactic and adhesion receptors between EMM and MM. **Bottom**: Next-generation sequencing studies show increased expression of free light-chains and CD70 in EMM cells and decreased expression of therapeutic targets such as CD38, SLAMF7, GPRC5D, and FCRH5. There was also enrichment of CD8+ T cells and NK cells within the EMM microenvironment. (Created using Biorender).

Extramedullary myeloma cells generally downregulate chemokine receptors (e.g., CXCR4, CCR1, CCR2) and adhesion molecules (e.g., CD56, CD177, VLA-4), while upregulating migration molecules (e.g., CD44, CD81, nestin) (Figure 1) [69]. CD56 is a membrane glycoprotein expressed by MM cells in approximately 65–80% of patients, but not normal plasma cells and is notably rarely expressed in plasma cell leukemia [61,70]. In MM cells, CD56 functions to anchor MM cells to the BM stroma, and an absence of CD56 has been associated with a higher incidence of extramedullary disease [62]. Analysis of paired bone marrow (BM) and extramedullary samples revealed that extramedullary multiple myeloma (MM) cells lacked CD56 expression, while BM myeloma cells exhibited varying levels of CD56 expression, further supporting the role of CD56 in extramedullary dissemination [61].

### 4.2. Insights from Molecular Studies

While the exact mechanisms underpinning the transformation of MM to EMM remain unknown, analysis of cytogenetic and gene expression profiles (GEPs) of paired samples from BM and extramedullary sites reveal spatial heterogeneity, suggesting that clonal selection with high-risk features may drive the development of EMM [71]. For example, a study found that BM samples had a higher frequency of del(13q14) and 14q32 rearrangements compared to EMM sites [72]. Another study investigating a small subset of 15 genes from the GEP-70 risk-stratification model [73] revealed significant differences in expression levels of 9/15 genes [74]. However, the role of these chromosomal abnormalities and genes in driving BM independence and extramedullary spread is yet to be determined. Indeed, the genetic and microenvironmental events leading to extramedullary disease escape are not fully understood. Although available cytogenetic studies suggest greater complexity in EMM compared to BM with enrichment of high-risk features such as t(4;14), del(17p), and chromosome 1 abnormalities [72,74,75,76,77,78,79,80,81,82,83,84,85,86,87], there is no unifying cytogenetic signature that predicts extramedullary disease. Studies also show that patients classified as high-risk by GEP have a higher likelihood of developing EMM, although GEP is not routinely performed in clinical practice currently [10]. A matched cohort study of patients with and without EMM implicated a younger age at diagnosis, 1q duplication, and t(4;14) as independent predictors of development of secondary EMM [47].

**Figure 2:**
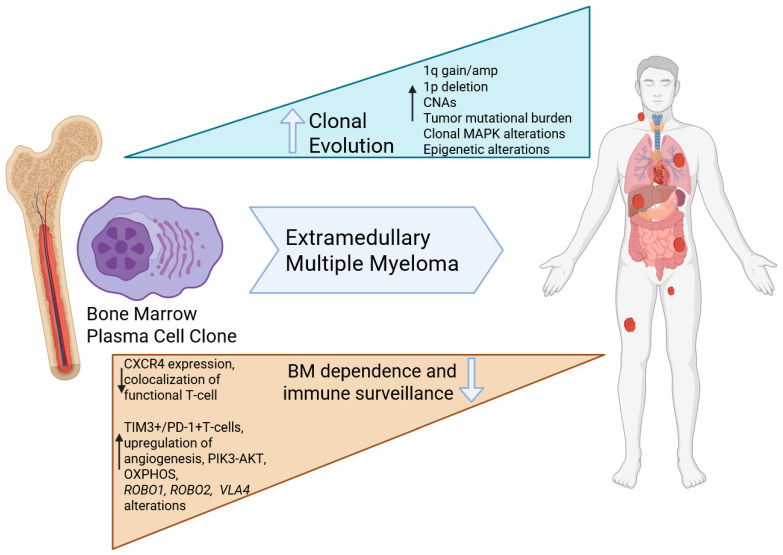
Current understanding of the molecular pathogenesis of EMM: Extramedullary tumors are enriched in MAPK alterations that are frequently clonal, and a complex genomic profile with higher mutational burden and copy number abnormalities. The microenvironment is enriched in exhausted T-cells and the plasma cells demonstrated altered metabolic programming and less dependence on the bone marrow (Created using Biorender).

A recent study performed FISH, whole-exome sequencing (WES), and bulk RNASeq sequencing of 14 EMM samples obtained from biopsies of soft tissue tumors, 8 paired and 6 unpaired samples obtain from the BM at the time of diagnosis, and 14 unpaired BM samples from RRMM patients without EMM [88]. Single-cell RNASeq and whole-exome sequencing were also performed on some samples. The study found that +1q21 was the most frequent aberration in EMM, present in 86%, followed by del(13q) and del(17p) at 57% and 43%, respectively [88] (Figure 2). In this cohort, mutations in the *MAPK* signaling pathway were found in 93% of EMM samples, with *KRAS* being the most frequently mutated gene in 71% of EMM samples [88]. Another whole-exome sequencing study of 18 EMM samples identified MAPK pathway mutations in 94% of samples, most commonly in *NRAS* (33%), *KRAS* (22%), and *BRAF* (22%) [89]. Mutations in the *RAS/RAF* pathway are common in MM (40–60% in both newly diagnosed and relapsed disease) and can be noted even in precursor conditions at lower frequencies [90,91,92]. In contrary to precursor conditions, the *RAS/RAF* mutations were more likely to be clonal in nature in EMM. Overall, EMM sites were noted to have a higher tumor mutational burden compared to bone marrow aspirates collected at the time of development of extramedullary disease [89]. Beyond the mutational profile, EMM was noted to be genomically complex, with a higher burden of copy number abnormalities compared to its bone marrow counterparts [89,93]. In particular, significant amplification peaks in CNAs were noted in 1q21+ and 1q21 gain/amplification, and *MAPK* pathway mutations co-occurred in a large proportion of EMM samples [88]. Importantly, co-occurrence of +1q21 and *KRAS* mutations was found to be associated with a significantly higher risk of soft tissue plasmacytoma development in the publicly available CoMMpass dataset (HR: 2.4, *p* = 0.011) [88]. Mutations in certain genes like *ROBO1* and *ROBO2*, which have a role in bone marrow stromal adhesion, were more frequent in EMM [89]. Murine models with infusion of *ROBO1* knockdown MM cell lines have demonstrated a higher degree of extramedullary spread [94]. *ROBO1* and *ROBO2* loss has also been demonstrated to increase cancer cell migration in other solid tumors, along with upregulation of the TGF-β and WNT pathways [95,96].

While the exact mechanisms underpinning the transformation of MM to EMM remain unknown, cytogenetic and next-generation sequencing studies have shown that EMM cells have a higher tumor mutational burden, greater copy number abnormalities (including +1q21), enrichment in del(13q) and del(17p), and mutations within the MAPK pathway. Conversely, FISH studies reveal a higher frequency of del(13q14) and 14q32 rearrangements in BM samples compared to EMM sites.

Recently, a few small studies have reported single-cell RNA sequencing data for EMM. A common theme of their findings is a preponderance of clonal plasma cells, paucity of the immune cell fraction, and predominantly exhausted cytotoxic T-cell subset with significant intra- and interpatient heterogeneity. Patients with EMM demonstrated a lower expression of CXCR4, supporting decreased BM homing as a mechanism of extramedullary dissemination. Additionally, EMM cells underexpress therapeutic targets such as CD38, SLAMF7, GPRC5D, and FCRH5, implicating antigen loss as a possible mechanism of resistance to monoclonal antibodies and T-cell-redirecting therapies. Notably, however, BCMA, the most prevalent target, maintained its expression levels [88] (Figure 1).

### 4.3. Insights from Immune Microenvironment Studies in EMM

Significant intralesional heterogeneity exists within EMM. A recent spatial transcriptomic study of EMM tumors demonstrated that exhausted TIM3+/PD-1+ T cells diffusely colocalized with MM cells, whereas functional and activated CD8+ T cells showed a focal infiltration pattern along with M1 macrophages in tumor-free regions. Additionally, there was a significant intralesional heterogeneity for CNAs and expression of relevant therapeutic targets like GPRC5D and TNFRSF17 [97]. Another single-cell RNA sequencing study of five EMM samples showed a relative paucity of immune cells in the EMM microenvironment, which were mainly comprised of CD8+ T cells (7%) and NK cells (1.8%) [88]. In contrast, a separate study utilizing multiplex immunofluorescence (mIF) to spatially immunoprofile the pre-CART microenvironment in EMM found that the proportion of CD8+ T cells was significantly reduced within tumor areas compared to adjacent normal areas. Additionally, the CD8+ T cells within the EMM tissue were exhausted, and expressed multiple inhibitory immune checkpoints including PD-1, LAG-3, and TIM-3 [98]. Single-cell RNASeq also showed significant interpatient heterogeneity of EMM cells, reinforcing the notion that multiple distinct molecular pathways can lead to the development of extramedullary disease [88]. Another single-cell RNASeq analysis of MM cells obtained from BM biopsies of nine RRMM and pleural effusion or ascites from four EMM patients identified differentially overexpressed genes involved in proliferation/cell-cycle progression, glycolysis, oxidative phosphorylation, proteasome, and antigen presentation genes in EMM versus higher levels of TNFa-induced NFkB pathway genes in BM-derived MM [83].

Finally, a single-cell RNA and ATAC sequencing study identified 68 “stromal interaction” genes overexpressed in MM cells co-cultured with BM stromal cells and significantly enriched in EMM obtained from malignant ascites and pleural effusions, suggesting that autonomous upregulation of these genes could contribute to BM stromal independence and survival outside the BM [65]. Functional annotation revealed that these genes play a role in cytokine/chemokine signaling, extracellular matrix organization, and cellular migration [65]. Ten out of these sixty-eight genes were independently prognostic of survival: five were associated with favorable survival (AKAP12, GADD45A, VCAN, FSCN1, FSTL1) and five with decreased survival (AIM2, ZEB2, IL6, ARAP3, PTK2). A summary measure of the expression of these 10 genes was used to develop an adverse stromal interaction (ASI+) signature. Patients with an ASI+ signature were noted to have increased rates of detectable CTCs at diagnosis. Additionally, ASI+ in newly diagnosed MM was associated with an increased prevalence of disseminated bone disease at diagnosis and higher rates of progressive bone and soft tissue disease over time [65].

Overall, EMM lesions are genomically complex and heterogeneous that are enriched in *MAPK* pathway mutations and copy number abnormalities, especially 1q gain/amplifications, and an immune microenvironment that is notable for exhausted effector T-cells. While no exclusive unifying marker for EMM biology has been identified, these recent biological insights have been crucial to improve our understanding of this aggressive entity.

## 5. Treatment and Prognosis of Multiple Myeloma and Extramedullary Multiple Myeloma in the Era of Novel Therapies

The past two decades have seen the approval of 16 new drugs, marking three treatment eras: (1) conventional chemotherapy (1950s–early 2000s), (2) novel therapies (early 2000s–early 2020s), and T-cell-redirecting therapies (late 2010s–present). This has led to significant improvements in OS for patients with MM [99,100]. A recent single-center retrospective study from Spain reported a quadrupling of median OS from 1980 to 2020 (22.4 to 103.6 months), driven largely by novel therapies including proteasome inhibitors (PIs), immunomodulatory drugs (IMiDs), and anti-CD38 monoclonal antibodies [101]. In contrast, survival improvements in EMM have been comparatively modest (summarized in Table 1). A retrospective study did not demonstrate any improvement in overall survival for patients with EMM who were diagnosed after 2010 compared to those diagnosed before 2010 [47].

### 5.1. Outcomes of Primary EMM in the Era of Novel Therapies

Primary EMM is defined here as EMM present at the initial diagnosis. A meta-analysis of eight trials of IMiD- or PI-based therapy between 2010 and 2018 included 12 patients with primary EMM, with a median OS of 70.1 months for EMM versus 79.9 months in patients without EMM [19]. A multi-center retrospective analysis of 226 patients treated across 11 European countries between 2010 and 2017 with novel therapies including anti-CD38 monoclonal antibodies pooled 92 patients with primary EMM and reported a median OS of 46.5 months [20]. In contrast, the median OS was not reached in the 38 patients with primary PSD that were included in the analysis [20]. The European Society for Blood and Marrow Transplantation (EBMT) published two registry analyses of patients with primary EMM who received upfront ASCT between 2003 and 2015, including approximately 85 and 139 patients (with some overlap). In the first study, the 4-year OS was 60% in patients with EMM alone and 46% in patients with both EMM and PSD [21]. In the second study, the 3-year OS was 58% in EMM versus 80.1% in patients without EMM or PSD [17]. A retrospective review by the Mayo Clinic for patients diagnosed between 2000 and 2021 reported that patients with primary EMM had an inferior median OS of 3.6 years compared to 7.1 years in a matched cohort without EMM, further supporting the notion that EMM is biologically distinct with an inferior prognosis [47]. A 2017 single-center retrospective analysis from South Korea compared 22 patients with primary EMM to 42 patients with primary PSD treated with PI and/or IMiD-based regimens between 2009 and 2016 [22]. The study reported a 2-year OS of 35.1% for EMM versus 52.6% in 42 patients with primary PSD [22]. A single-center Chinese study retrospectively identified 40 patients with primary EMM (and 28 patients with secondary EMM) in their cohort of 834 patients treated with PI or IMiD combinations and reported a median OS of 16.5 months in patients with EMM versus 40 months in patients without EMM or PSD [26]. In 2015, the Dana Farber Cancer Institute (DFCI) published a retrospective analysis of 55 patients with EMM (of which 13 were new diagnoses) treated with PI and/or IMiD-based therapies between 2005 and 2011 and reported a median OS of 49.2 months [27]. A single-center phase II trial at the Mayo Clinic included 174 patients with RRMM treated with pomalidomide and dexamethasone, of whom 3 had primary EMM and 13 had secondary EMM. The median OS was 16 months in patients with EMM and was not reached in patients without EMM [31]. A single-center retrospective analysis from the US evaluated 1304 patients with novel therapies including monoclonal antibodies, of whom 26 had primary EMM [18]. The median OS for primary EMM was 20 months versus 45 months for patients without EMM [18].

Lastly, a recent single-arm study of 10 patients with primary EMD (5 with PSD, 5 EMM) treated with selinexor in combination with bortezimib, lenalidomide, and dexamethasone reported an ORR of 100% with 1 CR (20%) and 2 VGPRs (40%) in the EMM group. At a median follow-up of 15 (range: 5–18) months, the median PFS and OS were not met for the entire cohort, and the 1-year PFS and OS rates were 60% and 90%, respectively [102].

### 5.2. Outcomes of Secondary EMM in the Era of Novel Therapies

Secondary EMM is defined here as development of EMM at disease relapse. A multi-center retrospective analysis of 226 patients treated across 11 European countries between 2010 and 2017 included 84 patients with secondary EMM treated with novel agents including anti-CD38 monoclonal antibodies [20]. A single-center retrospective study from Italy of 329 patients identified 103 patients with secondary EMD treated with PI, IMiDs, and/or conventional chemotherapy between 2000 and 2010. The 103 EMD patients comprised 43 EMM, 26 PCL, and 34 PSD. The median OS was 1.6 years in the EMM and PCL group versus 2.4 years in patients with PSD and 11 years in patients without EMD [24]. A long-term follow-up of eight clinical trials involving 117 patients treated with upfront bortezomib-based regimens with/without lenalidomide at the Dana-Farber Cancer Institute (DFCI) between 2003 and 2012 identified 19 patients with secondary EMM and 21 patients with PSD. The median OS was 0.9 years for secondary EMM compared to 2.5 years for PSD [28]. A single-center retrospective analysis from Germany included 351 patients with RRMM treated with PI and/or IMiD-based regimens between 2007 and 2010, of whom 24 had secondary EMM with a median OS of 7 months [30]. Finally, a single-center retrospective analysis from the US evaluated 1304 patients with novel therapies including monoclonal antibodies, of which 50 had secondary EMM [18]. The median OS of secondary EMM was 13 months versus 20 months for patients without EMM [18].

## 6. Treatment and Prognosis of Multiple Myeloma and Extramedullary Multiple Myeloma in the Era of T-Cell-Redirecting Therapies

Chimeric antigen receptor T-cell therapy (CART) targeting B-cell maturation antigen (BCMA) and bispecific T-cell engagers against BCMA and G-protein coupled receptor class C group 5 member D (GPRC5D) have emerged as highly effective options even in patients with triple-class and penta-refractory disease. Currently, there are two commercial BCMA-CART products available for RRMM in the United States: idacabtagene-vicleucel (ide-cel) and ciltacabtagene-autoleucel (cilta-cel). Ide-cel was approved based on a phase 2 KarMMa study, which demonstrated an overall response rate (ORR) of 73% and median PFS of 8.8 months [103]. Cilta-cel showed an ORR of 97.9% and median PFS of 34.9 months in the phase 1b/2 CARTITUDE-1 registration trial [104]. Additionally, there are three approved bispecific T-cell engagers. Teclistamab, a BCMAxCD3 bispecific antibody, was evaluated in the phase 1/2 MajesTEC-1 study, reporting an ORR of 63%, median PFS of 11.4 months, and median OS of 22.2 months [105]. Elranatamab, another BCMAxCD3 bispecific antibody, demonstrated an ORR of 61% and 15-month PFS of 50.9% in the phase 2 MagnetisMM-3 study [106]. Talquetamab, a GPRC5DxCD3 bispecific antibody, showed an ORR of 74% and median PFS of 11.9 months in the phase 1/2 MonumenTAL-1 study [107].

### 6.1. Outcomes of EMM in Patients Treated with Chimeric Antigen T-Cell Therapy (CART)

The registrational trials for ide-cel (KarMMa) and cilta-cel (CARTITUDE-1) included 50 (39%) and 13 (13%) patients with extramedullary disease (EMD), respectively (Table 2) [103,104]. Notably, the KarMMa study combined EMM and PSD together in their reporting. Distinguishing EMM from PSD remains important even in the era of cellular therapies as emerging data show that EMM is associated with significantly worse PFS and OS following CART compared to PSD [108,109]. For example, in a retrospective study, patients with PSD treated with either cilta-cel or ide-cel had similar survival outcomes to those without EMM: the median progression-free survival was 11.2 versus 13.6 months, respectively, and the median overall survival was not reached versus 27.5 months [109]. In contrast, patients with EMM had significantly worse outcomes compared to the BM-only group, with a median progression-free survival of 5.1 versus 13.6 months and a median overall survival of 12.2 versus 27.5 months [109].

Both trials (KarMMa and CARTITUDE-1) reported that patients with EMD had poorer outcomes compared to those without EMD, a finding that has been consistently supported by several subsequent retrospective analyses. A multi-center retrospective study in the US evaluated 351 RRMM patients treated with ide-cel. Among them, 84 patients had EMM. The Day 90 ORR for patients with EMM was 52%, compared to 82% in patients without EMM [16]. The median PFS and OS were 5.3 months and 14.8 months in patients with EMM versus 11.1 months and 26.9 months in patients without EMM, respectively [16]. Notably, EMM had the worse outcomes, with a median PFS of 5.3 months comapred to 9.2 months for PSD and 11.9 months for patients without EMM or PSD (*p* < 0.0001) [16]. A multi-center retrospective analysis published in 2024 included 152 RRMM patients, of whom 47 had EMM. In this study, 29% of patients received cilta-cel, while the remaining 79% patients received ide-cel [109]. The ORR was 58% in patients with EMM versus 96% in those without [109]. The median PFS and OS were also lower in EMM patients: 5.1 months versus 12.4 months for PFS, and 12.2 months versus 27.5 months for OS [109]. A recent multi-center study of 255 patients treated with cilta-cel, with 26% of patients having EMM, demonstrated that the presence of EMM was independently associated with an inferior PFS (HR of 1.96) and OS (HR of 1.88) compared to patients without EMM [131].

### 6.2. Predictors of Response to CART

Predictors of a poor response to CART include a high burden of extramedullary disease. One study found that patients with a lower disease burden (product of the two longest perpendicular diameters [SPD] < 50 cm^2^) achieved deep responses to CART, with two-thirds of patients achieving a complete radiographical response. In contrast, patients with higher tumor burdens (SPD ≥ 50 cm^2^) showed poor responses [108]. A recent study proposed a risk stratification model (MyCarE) to identify RRMM patients at a high risk of early progression. The model includes four risk factors: extramedullary disease with organ involvement (EMM), plasma cell leukemia (PCL), lenalidomide-refractory disease, and elevated serum ferritin levels at the time of lymphodepletion [110]. The risk of relapse or progression at 5 months varied dramatically based on these factors—only 7% of patients with no risk factors experienced progression, compared to 53% of patients who had all four risk factors [110]. Another study found that patients with higher proportions of exhausted CD8+ T cells and M2 macrophages at extramedullary tumor sites had poorer treatment outcomes [98]. Interestingly, levels of extramedullary tumor-infiltrating CARTs did not correlate with the durability of the response. In biopsy samples taken both after CAR T-cell therapy and during EMD progression, levels were low regardless of whether patients had achieved a durable response (remaining progression-free for at least 6 months) or experienced early progression (within 6 months) [98].

### 6.3. Toxicities and Patterns of Relapse After CART

Patients with EMM have comparable to slightly higher rates of cytokine release syndrome (CRS), immune-effector cell-associated neurotoxicity, and hematological toxicities [109]. The rate of CRS was 81% in EMM versus 78% without EMM (grade 3–5 CRS rates were 11% versus 3%). The rates of ICANS were 36% versus 25% (grade 3–4 ICANS rates were 6% versus 7%). Finally, hematologic toxicities were experienced by 74% versus 68% on day 30 (grades 3–5 in 48% versus 36%) and 43% versus 32% on day 90 (grades 3–5 10% versus 15%) [109].

The pattern of relapse after CART in EMM is heterogenous. Some studies show that patients experience bone marrow and extramedullary relapses at comparable rates [16,47,98,111]. However, other studies report that most EMM patients primarily relapse at extramedullary sites, with up to 42.9% maintaining minimal residual disease (MRD) negativity in their bone marrow assessments [108]. Among patients who experienced extramedullary relapse, most relapses occurred at the original disease sites rather than at new locations [132].

## 7. Outcomes of EMM in Patients Treated with Bispecific T-Cell Engagers (BiTEs)

In subgroup analyses of the MajesTEC-1 registrational trial for teclistamab, two cohorts were analyzed. Cohort A comprised triple-class-exposed patients with RRMM who had been previously treated with a PI, IMiD, and anti-CD38 therapy. Among these patients, 28 (17%) had extramedullary multiple myeloma with extraskeletal involvement (EMM). The ORR was notably lower in patients with EMM at 35.7%, compared to 68.6% in those without EMM (Table 2) [133]. Cohort C included more heavily pretreated patients who had received PI, IMiD, anti-CD38, and BCMA-directed therapy. In this group, 12 (30%) patients had EMM. Interestingly, these patients showed a higher ORR of 58.3%, exceeding the overall cohort’s response rate of 52.5% [130]. This finding was unexpected as these patients had previously relapsed after BCMA-directed therapy, though the small size of these subsets limits meaningful comparison. Several real-world studies demonstrated a consistently inferior efficacy of teclistamab in patients with EMM. First, a multi-center study from Germany including 43 patients with EMD, which reported an ORR of 37.2% versus 72.6% in patients with and without EMD, respectively [126]. The median PFS was 2.1 months in the EMD group but was not reached in those without EMD [126]. Next, a multi-center retrospective study from the US reported an ORR of 43% among 48 patients with EMM versus 58% in patients without EMM [127]. Finally, another multi-center US study of 45 EMM patients showed an ORR of 47% versus 80.3% in patients without EMM [134]. The PFS was significantly inferior in patients with EMM compared to those without EMM [HR 2.56 (95% CI: 1.37–4.76)] [134]. A recent encouraging report of the RedirecTT-1 study, assessing the combination of teclistamab with talquetamab (GRPC5D bispecific antibody) in RRMM, demonstrated an encouraging ORR of 61% in EMM. Additionally, the duration of response among responders was encouraging, with an 18-month PFS of 80% in the EMM cohort [135]. Elranatamab, another BCMA-directed BiTE, was studied in the phase II MagnetisMM-3, which included 39/123 patients with EMD (PSD or EMM) [106]. The ORR was 38.5% in the EMD group compared to 71.4% in patients without EMD. Among responders, the 15-month PFS was 77.9% in patients with EMD compared to 70.6% in the non-EMD cohort. Notably, very few of these patients were exposed to prior immune effector therapies, but these exciting results lay down the benchmark for future trials to improve upon.

## 8. Conclusions

EMM continues to present a significant therapeutic challenge, even in the context of novel therapies as well as T-cell-redirecting therapies such as CARTs and bispecific antibodies. The greater genomic complexity in EMM with enrichment of high-risk features such as t(4;14), del(17p), and chromosome 1 abnormalities [72,74,75,76,77,78,79,80,81,82,83,84,85,86,87,136] contributes to a poor prognosis. Additionally, evidence suggests that EMM cells downregulate therapeutic targets like CD38, SLAMF7, GPRC5D, and FCRH5, and studies into the immune microenvironment of EMM reveal a paucity of the immune cell fraction and predominantly exhausted cytotoxic T-cell subset. While these findings indicate that antigen loss and poor T-cell fitness may contribute to resistance against monoclonal antibodies and T-cell-redirecting therapies, it is important to recognize that these small studies need to be validated in larger cohorts. Given the limited efficacy of existing treatment options in EMM, all available therapeutic options need to be explored, including the use of alkylating agents if not used in the recent lines of therapy. Clinical trials incorporating novel agents are the need of the hour for EMM.

## 9. Future Directions

Extramedullary spread represents a watershed moment in the disease trajectory of patients with MM. The past two decades have witnessed remarkable progress in our understanding of MM biology, leading to the development and approval of numerous novel therapeutics including T-cell-redirecting therapies. With the improving overall survival of patients, we are encountering an increasing proportion of heavily pretreated patients, who are enriched in the presence of extramedullary disease. Recent clinical trial enrollment data demonstrate that 20–30% of patients can have EMM at the time of enrollment. Yet, dedicated clinical trials for EMM are not yet a reality. The recent report of the EMN012 study, enrolling patients withprimary plasma cell leukemia, should serve as a template for future cooperative group studies for this aggressive entity [137]. The sustained responses with the teclistamab and talquetamab combination for EMM are encouraging signs. BRAF/MEK inhibition is currently being evaluated in clinical trials for BRAF^V600E^ mutated MM. A phase 2 trial of encorafenib and binimetinib reported an ORR of 83.3%, an mPFS of 5.6 months, and a 2y OS of 55% in heavily pretreated RRMM patients, with a median of five prior lines of therapy [138]. Given the significantly higher prevalence of activating MAPK pathway mutations in EMM, targeting MEK ± BRAF in combination with other effective therapies may offers a promisingc strategy. A retrospective review of 51 patients treated with single-agent MEK inhibitor trematinib included 11 patients with extramedullary disease. Of the 24 patients with at least one FDG-avid focal lesion on PET imaging before starting trametinib, 15 experienced an >50% reduction in the number of focal lesions, with 10 achieving a PET complete response, suggesting that MEK inhibition has activity in MM, although this needs to be studied further in extramedullary sites of disease [139].

Novel combinations, especially those incorporating newer molecules like the CelMod mezigdomide in combination with an MEK inhibitor, demonstrated impressive ORRs, but whether these are sustained remains to be seen [140]. Biology-driven novel combination therapies tested in a clinical trial setting are key to improving outcomes for patients with EMM.

## Figures and Tables

**Table 1:** Incidence of EMM and outcomes in the era of conventional chemotherapy and novel therapies.

Study Type	Year Published (Time Period)	Imaging Used to Assess for EMD	Population	Type of EM Involvement	Incidence of EMM	Treatments	PFS	OS	Ref
Single-center retrospective analysis (USA)	2022 (1970–2018)	Not reported	1304 patients (256 EMD)	PSD (1º): 230 (17.6%) EMM (1º): 26 (2%) PSD (2º): 142 (14.6%) EMM (2º): 50 (5.1%)	1º: 2% 2º: 5.1%	Chemotherapy (*n* = 873); PI (*n* = 180); IMiD (*n* = 70); PI + IMiD (*n* = 150); Monoclonal antibody (*n* = 16) ASCT (*n* = 413)	Not reported	mOS (1º): 20 (EMM) vs. 44 (PSD) vs. 45 (no EMM) months mOS (2º): 13 (EMM) vs. 14 (PSD) vs. 20 (no EMM) months	[18]
Meta-analysis of 8 trials: GIMEMA-MM-05-05, GIMEMA-MM-03-05, RV-MM-PI-209, RV-MM-EMN-441, EMN01, MMY2069, IST-CAR-506, IST-CAR-561	2020 (2010–2018)	Skeletal survey, MRI, or CT	2322 NDMM (267 EMD)	PSD (1º): 243 (10.4%) EMM (1º): 12 (0.5%) Unclassified: 12 (0.5%)	1º: 0.5%	IMiD backbone (*n* = 166), PI backbone (*n* = 66), IMiD + PI backbone (*n* = 35) followed by ASCT (*n* = 155)	mPFS: 24.3 (PSD) vs. 26.1 (EMM) vs. 25.2 (no EMD) months	mOS: 67.3 (PSD) vs. 70.1 (EMM) vs. 79.9 (no EMD) months	[19]
Multi-center retrospective analysis (11 European countries)	2020 (2010–2017)	PET/CT (*n* = 50), MRI (*n* = 35), CT (*n* = 133)	226 ND/RRMM with EMD	PSD (1º): 38 (16.8%) EMM (1º): 92 (40.7%) PSD (2º): 12 (5.3%) EMM (2º): 84 (37.2%)	Not reported	PI, IMiD PI + IMiD Monoclonal antibody	mPFS (1º): 51.7 (PSD) vs. 38.9 (EMM) months mPFS (2º): 20.9 (PSD) vs. 11.4 (EMM) months	mOS (1º): NR (PSD) vs. 46.5 (EMM) months; mOS (2º): 39.8 (PSD) vs. 13.6 (EMM) months	[20]
EBMT registry analysis	2019 (2003–2015)	Not reported	488 NDMM with EMD	PSD (1º): 76% EMM (1º): 18%	Not reported	Upfront ASCT (single or tandem or auto-allo) within 12 months of diagnosis	4y PFS: 44% (PSD) vs. 39% (EMM)	4y OS: 72% (PSD) vs. 60% (EMM) vs. 46% (both)	[21]
EBMT registry analysis	2018 (2005–2014)	Not reported	3744 NDMM (682 EMD)	PSD (1º): 543 (14.5%) EMM (1º): 139 (3.7%)	1º: 3.7%	Upfront ASCT (single or tandem) within 12 months of diagnosis	3y PFS: 50% (PSD) vs. 39.9% (EMM) vs. 47.9% (no EMD)	3y OS: 77.7% (PSD) vs. 58% (EMM) vs. 80.1% (no EMM)	[17]
Single-center retrospective analysis (South Korea)	2017 (2009–2016)	PET/CT	64 NDMM with EMD	PSD (1º): 42 (65.6%) EMM (1º): 22 (34.4%)	Not reported	Vd (*n* = 7), Td (*n* = 23), VTd (*n* = 11), VMP (*n* = 16), MP (*n* = 6); followed by ASCT (*n* = 28)	mPFS: 16.1 (PSD) vs. 16 (EMM) months	2y OS (1º): 52.6% (PSD) vs. 35.1% (EMM)	[22]
Single-center retrospective analysis (India)	2017 (Not reported)	PET/CT, MRI	271 NDMM (44 EMD)	PSD (1º): 30 (11.1%); EMM (1º): 8 (3%); PSD + EMM (1º): 6 (2.2%)	1º: 5.2%	Novel agents (*n* = 27), Conventional chemo (*n* = 17); followed by ASCT (*n* = 44)	mPFS: 18 (all EM) vs. 44 (no EM) months	mOS: 32 (all EM) vs. 100 (no EM) months	[23]
Single-center retrospective analysis (Italy)	2016 (2000–2010)	XR, CT, MRI	329 patients (103 with secondary EMD)	PSD (2º): 34 (10.3%) EMM (2º): 43 (13.1%) PCL (2º): 26 (7.9%)	2º: 13.1%	Not reported specifically for EMM (chemotherapy, bortezomib, lenalidomide, thalidomide)	Not reported	mOS: 2.4 (PSD) vs. 1.6 (EMM + PCL) vs. 11 (no EMM) years	[24]
Multi-center phase 2 clinical trial (Japan)	2016	Not reported	36 RRMM (5 EMM)	EMM-B: 4 EMM: 1 (2.8%)	2.8%	Pomalidomide + Dex	Not reported specifically for EMM but none of the EMM patients achieved ≥ PR		[25]
Single-center retrospective analysis (China)	2015 (1993–2013)	XR, US, CT	834 patients (68 EMD)	EMM (1º): 40 (4.8%) EMM (2º): 28 (3.4%)	1º: 4.8% 2º: 3.4%	IMiD backbone (*n* = 11), PI backbone (*n* = 17), Alkylating agent (*n* = 4), Dexamethasone-based (*n* = 8) followed by ASCT (*n* = 5)	mTTP: 11.5 (EMM) vs. 25 (no EMM) months	mOS: 16.5 (EMM) vs. 40 (no EMM) months	[26]
Single-center retrospective analysis (USA; DFCI)	2015 (2005–2011)	Not reported	663 patients treated with ASCT (55 EMD)	EMM (1º): 13 (2%) EMM (2º): 42 (6.3%)	1º: 2% 2º: 6.3%	Td (*n* = 25), RVd (*n* = 23), Vd (*n* = 15); ASCT (*n* = 55); AlloSCT (*n* = 15)	Not reported	mOS: 49.2 months (from MM diagnosis); 15.6 months (from EMM diagnosis)	[27]
Single-center retrospective analysis (USA; DFCI/MGH)	2015 (2003–2012)	CT, PET/CT, MRI	117 patients (40 with EMD at diagnosis; 40 at relapse)	PSD (1º): 38 (32.5%) EMM (1º): 2 (1.7%); PSD (2º): 21 (17.9%) EMM (2º): 8 (6.8%) PSD + EMM (2º): 11 (9.4%)	1º: 1.7% 2º: 16.2%	8 clinical trials of upfront bortezomib-based regimens with/without lenalidomide; followed by ASCT (*n* = 57) or AlloSCT (*n* = 4)	Not reported	2º: mOS: 2.47 (PSD) vs. 0.9 (EMM) years	[28]
Single-center retrospective analysis (Czech Republic)	2013 (2005–2008)	US, CT, MRI	226 RRMM (55 with EMD at relapse)	PSD (2º): 23 (10.2%) EMM (2º): 32 (14.2%)	2º: 14.2%	Treatment at time of EM relapse not reported	mTTP: 5.4 months	mOS: 38 (EM) vs. 109 (no EM) months; 30 (EMM) vs. 45 (PSD) months	[29]
Single-center retrospective analysis (Germany)	2012 (2007–2010)	Not reported	357 RRMM (24 EMD)	EMM (2º): 24 (6.7%)	2º: 6.7%	RT (*n* = 16), ASCT (*n* = 6), velcade (*n* = 16), lenalidomide (*n* = 12), thalidomide (*n* = 8)	mPFS: 2 months	mOS: 7 months	[30]
Single-center retrospective analysis (USA; UAMS)	2012 (2000–2010)	PET/CT	1965 patients (101 EMD)	EMM (1º): 66 (3.4%) EMM (2º): 35 (1.8%)	1º: 3.4% 2º: 1.8%	ASCT as part of total therapy and non-total therapy protocols	5y PFS (1º): 21% (EMM) vs. 50% (no EMM)	5y OS (1º): 31% (EMM) vs. 59% (no EMM)	[10]
Single-center phase 2 clinical trial (USA; Mayo Clinic)	2011 (2007–2010)	PET/CT, CT, MRI	174 RRMM (16 EMM)	EMM (1º): 3 (1.7%) EMM (2º): 13 (7.5%)	1º: 1.7% 2º: 7.5%	Pomalidomide + Dex	Not reported	mOS: 16 (EMM) vs. NR (no EMM) months	[31]
Single-center retrospective analysis (Italy)	2009 (1971–2007)	XR, CT, MRI	1003 MM patients (132 with EMM; 76 at diagnosis, 56 at relapse)	PSD (1º): ~65 (6.5%) EMM (1º): ~11 (1.1%); PSD (2º): ~40 (4%) EMM (2º): ~16 (1.6%); PCL (2º): 9 (0.9%)	1º: ~1.1% 2º: ~1.6%	1971–1993: conventional-dose chemotherapy; 1994–1999: ASCT < 65yo; 2000–2007: PI, IMiD-based	mPFS: 18 (all EMM) vs. 30 (no EMM) months	mOS: 36 (all EMM) vs. 43 (no EMM) months	[11]
Multi-center retrospective analysis (7 centers in the Netherlands)	2008	Not reported	172 patients who received sequential auto-alloSCT	EMM (2º): 11 (6.4%)	2º: 6.4%	Donor lymphocyte infusions (*n* = 3), thalidomide (*n* = 6), bortezomib (*n* = 2), dexamethasone (*n* = 2), RT (*n* = 4)	mPFS: 2.4 years (not significantly different between EMM and no EMM)	1y OS: 61% (EMM) vs. 73% (no EM)	[32]
Multi-center retrospective analysis (Grupo Espanol de Mieloma)	2006 (1999–2004)	Imaging only if symptomatic	70 patients who received alloSCT	EMM (2º): 10 (14.3%)	2º: 14.3%	Not reported	Not reported	Not reported	[33]

EMM: extramedullary multiple myeloma; EMD: extramedullary disease including EMM and paraskeletal disease (PSD) when the study does not specify. HR: hazard ratio; OS: Overall Survival; ORR: overall response rate; mPFS: median progression-free survival; PCL: plasma cell leukemia, PD: progressive disease; PSD: paraskeletal disease; RRMM: relpased/refractory multiple myeloma; TTP: time-to-progression; 1º: primary EMM, 2º: secondary EMM.

**Table 2:** Summary of outcomes in the era of T-cell-redirecting therapies.

Study Type	Year Published (Time Period)	Population	Type of T-Cell-Redirecting Therapy	Lines of Therapy Prior to CART/BiTe	Efficacy in EMM	Reference
Multi-center retrospective analysis (USA)	2024 (data cutoff: 2023)	152 RRMM (47 EMM)	Cilta-cel Ide-cel	Median: 6; range: 4–15	ORR: 58% (EMM) vs. 96% (no EMM); mPFS: 5.1 (EMM) vs. 12.4 (no EMM) months; mOS: 12.2 (EMM) vs. 27.5 (no EMM) months	[109]
Multi-center retrospective analysis (international)	2024 (not reported)	269 RRMM (112 EMM)	Cilta-cel Ide-cel Investigational	Median: 6; range: 6–7	EMM predicts for early relapse/progression post-CART (HR: 1.92)	[110]
Single-center retrospective study (USA; Moffitt)	2024 (2021–2023)	116 RRMM (20 EMM; 10 PSD; 7 EMM + PSD)	Cilta-cel Ide-cel	Median: 6; range: 4–9	mPFS: 15.7 months (all EMM); mOS: 3.1 months (all EMM)	[111]
Meta-analysis of BiTEs vs. CARTs	2023 (not reported)	172 RRMM with EMM (type of EMM not specified)	CART	Not reported	ORR: 77% (EMM)	[112]
Single-center retrospective study (USA; Mayo Clinic)	2023 (2000–2021)	299 RRMM patients with EMM; EMM (1º): 95; EMM (2º): 204	Cilta-cel Ide-cel Investigational	Median: 4; range: 4–8	ORR: 75%; mPFS: 4.9 months	[47]
Single-center retrospective study (USA)	2023 (2017–2023)	134 RRMM; EMM (1º): 34; PSD (1º): 25; EMM (2º): 13	Cilta-cel Ide-cel Investigational	Not Reported	HR for PFS of 1º EMM vs. no EMM: 1.87; HR for OS of 1º EMM vs. no EMM: 3.78	[108]
Multi-center phase 2 clinical trial (CARTITUDE-2C)	2023 (data cutoff: 2021)	20 RRMM (cohort C) (5 EMM; type of EMM not specified)	Cilta-cel	Median: 8; range: 4–13	ORR: 60% (EMM)	[113]
Multi-center phase 1/2b clinical trial (CARTITUDE-1)	2021 (2018–2019)	113 RRMM (13 EMM; type of EMM not specified)	Cilta-cel	Median: 6; range (4–8)	mDOR: 12.9 months (EMM) vs. 23.3 months (all patients)	[104]
Multi-center retrospective analysis (USA)	2024 (2021–2023)	351 RRMM (84 EMM)	Ide-cel	Median: 6; range: 5–8	ORR: 52% (EMM) vs. 82% (no EMM); mPFS: 5.3 (EMM) vs. 11.1 (no EMM) months; mOS: 14.8 (EMM) vs. 26.9 (no EMM) months	[16]
Multi-center phase 2 clinical trial (KarMMa)	2021 (2017–2020)	128 RRMM (50 EMM; type of EMM not reported)	Ide-cel	Median: 6; range: 3–16	ORR of EMM worse than no EMM (no specifics)	[103]
Multi-center phase 1 clinical trial (CRB-401)	2019 (2016–2018)	33 RRMM (9 EMM; type of EMM not reported)	Ide-cel	Median: 7; range: 3–14	ORR: 89% (EMD)	[114]
Meta-analysis of 17 trials	2023	723 RRMM	Investigational	Median: 5	RR for ORR of EMM vs. no EMD: 0.97; RR for PFS of EMM vs. no EMM: 1.44; RR for OS of EMM vs. no EMM: 1.96	[115]
Single-center phase 1/2a clinical trial (China)	2024 (2018–2022)	16 RRMM (6 EMD; 3 had EMM without medullary disease; type of EMM not reported)	Investigational (bispecific anti-BCMA and anti-CS1 CART)	Median: 4; range: 2–8	ORR: 0% (in 3 patients with EMM without medullary disease)	[116]
Single-center prospective cohort study (China)	2024 (2017–2023)	31 RRMM patients with EMM	Investigational (anti-BCMA and anti-CD19 CART)	Median: 4; range: 3–12	ORR: 90% (medullary and serological); 64.5% (imaging); mPFS: 5 months; mOS: 9.7 months	[98]
Single-center phase 2 clinical trial (China)	2022 (2017–2021)	69 RRMM (15 EMM; type of EMM not reported)	Investigational (anti-BCMA and anti-CD19 CART)	Median: 4; range (2–17)	ORR: 80%; mPFS: 8.3 (EM M) vs. 21.4 (no EMM) months; mOS: 12.3 (EMM) vs. NR (no EMM) months	[117]
Single-center phase 1 clinical trial (China)	2021 (2018–2021)	61 RRMM (7 EMM, 10 PSD, 10 EMM + PSD)	Investigational (anti-BCMA CART)	Median: 3; range (3–9)	CR: 59% (EMM) vs. 81% (no EMM); 1 yr PFS: 34.4% (EMM) vs. 60.2% (no EMM)	[118]
Single-center retrospective analysis of 2 clinical trials (China)	2021 (2017–2021)	60 RRMM (25 EMM/PCL; 35 without EMM)	Investigational: 25 (CT103A: 7, murine anti-BCMA CART: 18)	Median: 4; range: 3–11	ORR: 84% (EMM) vs. 94.4% (no EMM); mPFS: 121 (EMM) vs. 361 (no EMM) days; mOS: 248 (EMM) vs. 1024 (no EMM) days	[119]
Single-center phase 1 clinical trial (China)	2021 (2017–2019)	30 RRMM (14 EMM/PCL)	Investigational (murine anti-BCMA CART)	Median 4; range: 3–11	ORR: 92.8% (EMM/PCL)	[120]
Single-center phase 1 clinical trial (China)	2021 (2018–2020)	18 RRMM (4 EMM, 1 PCL)	Investigational (CT103A)	Median: 4; range: 3–6	ORR: 100%; 1-yr PFS: 20% (EMM) vs. 79.1% (no EMM); 1-yr OS: 60% (EMM) vs. 79.1% (no EMM)	[121]
Single-center phase 1 clinical trial (China)	2021 (2019–2021)	23 RRMM (7 PSD, 1 EMM, 1 unspecified)	Investigational (bispecific anti-BCMA and anti-CD38 CART)	Median: 4; range (2–9)	ORR: 89% (EMM)	[122]
Single-center phase 1 clinical trial (USA, UPenn)	2019 (2015–2018)	25 RRMM (7 EMD; type of EMD not reported)	Investigational (anti-BCMA CART)	Median: 7; range: 3–13	ORR: 57% (EMM)	[123]
Multi-center phase 1 clinical trial (China)	2019 (2017–2018)	17 RRMM (3 EMM, 2 PSD)	Investigational (LCAR-B38M)	Median: 4; range: 3–11	ORR: 100% (EMM)	[124]
Single-center phase 1 clinical trial (USA; NIH)	2018 (not reported)	16 RRMM (1 EMM)	Investigational (anti-BCMA CART)	16 (EMM)	ORR: 100% (EMM)	[125]
Multi-center retrospective analysis (Germany)	2024 (2022–2024)	123 RRMM (43 EMM; type of EMM not specified)	Teclistamab	Median: 6; range: 3–14	ORR: 37.2% (EMM) vs. 72.6% (no EMM); mPFS: 2.1 months (EMM) vs. NR (no EMM)	[126]
Multi-center retrospective analysis (USA)	2024 (2023–2024)	110 RRMM (48 EMM)	Teclistamab	Median: 6; range: 3–13	ORR: 43% (EMM) vs. 58% (no EMM)	[127]
Multi-center retrospective analysis (USA)	2024 (2022–2023)	106 RRMM (45 EMM)	Teclistamab	Median: 6; range: 4–18	ORR: 47% (EMM) vs. 80.3% (no EMM); HR for PFS of EMM: 2.56	[109]
Single-center retrospective study (USA; Mayo Clinic)	2023 (2000–2021)	299 RRMM patients with EMM; EMM (1º): 95 EMM (2º): 204	BiTE: 12 (BCMAxCD3: 10, GPRC5DxCD3: 1, FCRH5xCD3: 1)	Median: 5; range: 4–8	ORR: 59%; mPFS: 3.9 months	[47]
Meta-analysis of BiTE vs. CARTs	2023 (not reported)	106 RRMM with EMM (type of EMM not specified)	BiTE (teclistamab, talquetamab)	Not reported	ORR: 48% (EMM)	[112]
Single-center retrospective study (USA)	2023 (not reported)	7 RRMM (2 EMM; type of EMM not specified)	Teclistamab	Median: 4; range: 4–7	ORR: 0% (both patients had PD prior to second cycle)	[128]
Multi-center phase 1/2 clinical trial (MajesTEC-1; Cohort A)	2022 (2020–2022)	165 RRMM (28 EMM)	Teclistamab	Median: 5; range: 2–14	ORR: 35.7% (EMM) vs. 68.6% (no EMM)	[129]
Multi-center phase 1/2 clinical trial (MajesTEC-1; Cohort C)	2022 (2020–2022)	40 RRMM previously treated with PI, IMiD, anti-CD38, and BCMA-targeted therapies (12 EMM)	Teclistamab	Median: 6; range: 3–14	ORR: 58.3% (EMM) vs. 52.5% (all patients)	[130]

BiTe: Bispecific T-cell engagers; CAR-T: chimeric antigen receptor T-cell therapy; DOR: duration of response; EMM: extramedullary multiple myeloma; HR: hazard ratio; mPFS: median progression-free survival; PCL: plasma cell leukemia, PD: progressive disease; PSD: paraskeletal disease; ORR: overall response rate; RRMM: relpased/refractory multiple myeloma; 1º: primary EMM, 2º: secondary EMM.

## References

[1] Hideshima T., Mitsiades C., Tonon G., Richardson P.G., Anderson K.C. (2007). Understanding multiple myeloma pathogenesis in the bone marrow to identify new therapeutic targets. Nat. Rev. Cancer.

[2] Barwick B.G., Gupta V.A., Vertino P.M., Boise L.H. (2019). Cell of Origin and Genetic Alterations in the Pathogenesis of Multiple Myeloma. Front. Immunol..

[3] Moser-Katz T., Joseph N.S., Dhodapkar M.V., Lee K.P., Boise L.H. (2021). Game of Bones: How Myeloma Manipulates Its Microenvironment. Front. Oncol..

[4] Terpos E., Ntanasis-Stathopoulos I., Gavriatopoulou M., Dimopoulos M.A. (2018). Pathogenesis of bone disease in multiple myeloma: From bench to bedside. Blood Cancer J..

[5] Rajkumar S.V. (2016). Updated Diagnostic Criteria and Staging System for Multiple Myeloma. Am. Soc. Clin. Oncol. Educ. Book.

[6] Fernández de Larrea C., Kyle R., Rosiñol L., Paiva B., Engelhardt M., Usmani S., Caers J., Gonsalves W., Schjesvold F., Merlini G. (2021). Primary plasma cell leukemia: Consensus definition by the International Myeloma Working Group according to peripheral blood plasma cell percentage. Blood Cancer J..

[7] de Wergifosse M., Champagne B., Ito S., Fukuda K., Nakano M. (2016). Challenging compounds for calculating molecular second hyperpolarizabilities: The triplet state of the trimethylenemethane diradical and two derivatives. Phys. Chem. Chem. Phys..

[8] Fernandez de Larrea C., Kyle R.A., Durie B.G., Ludwig H., Usmani S., Vesole D.H., Hajek R., San Miguel J.F., Sezer O., Sonneveld P. (2013). Plasma cell leukemia: Consensus statement on diagnostic requirements, response criteria and treatment recommendations by the International Myeloma Working Group. Leukemia.

[9] Labopin M., Ruggeri A., Gorin N.C., Gluckman E., Blaise D., Mannone L., Milpied N., Yakoub-Agha I., Deconinck E., Michallet M. (2014). Cost-effectiveness and clinical outcomes of double versus single cord blood transplantation in adults with acute leukemia in France. Haematologica.

[10] Usmani S.Z., Heuck C., Mitchell A., Szymonifka J., Nair B., Hoering A., Alsayed Y., Waheed S., Haider S., Restrepo A. (2012). Extramedullary disease portends poor prognosis in multiple myeloma and is over-represented in high-risk disease even in the era of novel agents. Haematologica.

[11] Varettoni M., Corso A., Pica G., Mangiacavalli S., Pascutto C., Lazzarino M. (2010). Incidence, presenting features and outcome of extramedullary disease in multiple myeloma: A longitudinal study on 1003 consecutive patients. Ann. Oncol..

[12] Wali A., Kumar A.M.V., Hinderaker S.G., Heldal E., Qadeer E., Fatima R., Ullah A., Safdar N., Yaqoob A., Anwar K. (2017). Pre-treatment loss to follow-up among smear-positive TB patients in tertiary hospitals, Quetta, Pakistan. Public Health Action.

[13] Weinstock M., Ghobrial I.M. (2013). Extramedullary multiple myeloma. Leuk. Lymphoma.

[14] Banerjee R., Cicero K.I., Lee S.S., Cowan A.J. (2023). Definers and drivers of functional high-risk multiple myeloma: Insights from genomic, transcriptomic, and immune profiling. Front. Oncol..

[15] Caers J., Paiva B., Zamagni E., Leleu X., Bladé J., Kristinsson S.Y., Touzeau C., Abildgaard N., Terpos E., Heusschen R. (2018). Diagnosis, treatment, and response assessment in solitary plasmacytoma: Updated recommendations from a European Expert Panel. J. Hematol. Oncol..

[16] Zanwar S., Sidana S., Shune L., Puglianini O.C., Pasvolsky O., Gonzalez R., Dima D., Afrough A., Kaur G., Davis J.A. (2024). Impact of extramedullary multiple myeloma on outcomes with idecabtagene vicleucel. J. Hematol. Oncol..

[17] Gagelmann N., Eikema D.J., Iacobelli S., Koster L., Nahi H., Stoppa A.M., Masszi T., Caillot D., Lenhoff S., Udvardy M. (2018). Impact of extramedullary disease in patients with newly diagnosed multiple myeloma undergoing autologous stem cell transplantation: A study from the Chronic Malignancies Working Party of the EBMT. Haematologica.

[18] Jiménez-Segura R., Rosiñol L., Cibeira M.T., Fernández de Larrea C., Tovar N., Rodríguez-Lobato L.G., Bladé E., Moreno D.F., Oliver-Caldés A., Bladé J. (2022). Paraskeletal and extramedullary plasmacytomas in multiple myeloma at diagnosis and at first relapse: 50-years of experience from an academic institution. Blood Cancer J..

[19] Montefusco V., Gay F., Spada S., De Paoli L., Di Raimondo F., Ribolla R., Musolino C., Patriarca F., Musto P., Galieni P. (2020). Outcome of paraosseous extra-medullary disease in newly diagnosed multiple myeloma patients treated with new drugs. Haematologica.

[20] Beksac M., Seval G.C., Kanellias N., Coriu D., Rosinol L., Ozet G., Goranova-Marinova V., Unal A., Bila J., Ozsan H. (2020). A real world multicenter retrospective study on extramedullary disease from Balkan Myeloma Study Group and Barcelona University: Analysis of parameters that improve outcome. Haematologica.

[21] Gagelmann N., Eikema D.J., Koster L., Caillot D., Pioltelli P., Lleonart J.B., Remenyi P., Blaise D., Schaap N., Trneny M. (2019). Tandem Autologous Stem Cell Transplantation Improves Outcomes in Newly Diagnosed Multiple Myeloma with Extramedullary Disease and High-Risk Cytogenetics: A Study from the Chronic Malignancies Working Party of the European Society for Blood and Marrow Transplantation. Biol. Blood Marrow Transplant..

[22] Batsukh K., Lee S.E., Min G.J., Park S.S., Jeon Y.W., Yoon J.H., Cho B.S., Eom K.S., Kim Y.J., Kim H.J. (2017). Distinct Clinical Outcomes between Paramedullary and Extramedullary Lesions in Newly Diagnosed Multiple Myeloma. Immune Netw..

[23] Kumar L., Gogi R., Patel A.K., Mookerjee A., Sahoo R.K., Malik P.S., Sharma A., Thulkar S., Kumar R., Biswas A. (2017). Multiple myeloma with extramedullary disease: Impact of autologous stem cell transplantation on outcome. Bone Marrow Transplant..

[24] Mangiacavalli S., Pompa A., Ferretti V., Klersy C., Cocito F., Varettoni M., Cartia C.S., Cazzola M., Corso A. (2017). The possible role of burden of therapy on the risk of myeloma extramedullary spread. Ann. Hematol..

[25] Ichinohe T., Kuroda Y., Okamoto S., Matsue K., Iida S., Sunami K., Komeno T., Suzuki K., Ando K., Taniwaki M. (2015). A multicenter phase 2 study of pomalidomide plus dexamethasone in patients with relapsed and refractory multiple myeloma: The Japanese MM-011 trial. Exp. Hematol. Oncol..

[26] Deng S., Xu Y., An G., Sui W., Zou D., Zhao Y., Qi J., Li F., Hao M., Qiu L. (2015). Features of extramedullary disease of multiple myeloma: High frequency of p53 deletion and poor survival: A retrospective single-center study of 834 cases. Clin. Lymphoma Myeloma Leuk..

[27] Weinstock M., Aljawai Y., Morgan E.A., Laubach J., Gannon M., Roccaro A.M., Varga C., Mitsiades C.S., Paba-Prada C., Schlossman R. (2015). Incidence and clinical features of extramedullary multiple myeloma in patients who underwent stem cell transplantation. Br. J. Haematol..

[28] Varga C., Xie W., Laubach J., Ghobrial I.M., O’Donnell E.K., Weinstock M., Paba-Prada C., Warren D., Maglio M.E., Schlossman R. (2015). Development of extramedullary myeloma in the era of novel agents: No evidence of increased risk with lenalidomide-bortezomib combinations. Br. J. Haematol..

[29] Pour L., Sevcikova S., Greslikova H., Kupska R., Majkova P., Zahradova L., Sandecka V., Adam Z., Krejci M., Kuglik P. (2014). Soft-tissue extramedullary multiple myeloma prognosis is significantly worse in comparison to bone-related extramedullary relapse. Haematologica.

[30] Rasche L., Bernard C., Topp M.S., Kapp M., Duell J., Wesemeier C., Haralambieva E., Maeder U., Einsele H., Knop S. (2012). Features of extramedullary myeloma relapse: High proliferation, minimal marrow involvement, adverse cytogenetics: A retrospective single-center study of 24 cases. Ann. Hematol..

[31] Short K.D., Rajkumar S.V., Larson D., Buadi F., Hayman S., Dispenzieri A., Gertz M., Kumar S., Mikhael J., Roy V. (2011). Incidence of extramedullary disease in patients with multiple myeloma in the era of novel therapy, and the activity of pomalidomide on extramedullary myeloma. Leukemia.

[32] Minnema M.C., van de Donk N.W., Zweegman S., Hegenbart U., Schonland S., Raymakers R., Zijlmans J.M., Kersten M.J., Bos G.M., Lokhorst H.M. (2008). Extramedullary relapses after allogeneic non-myeloablative stem cell transplantation in multiple myeloma patients do not negatively affect treatment outcome. Bone Marrow Transplant..

[33] Perez-Simon J.A., Sureda A., Fernandez-Aviles F., Sampol A., Cabrera J.R., Caballero D., Martino R., Petit J., Tomas J.F., Moraleda J.M. (2006). Reduced-intensity conditioning allogeneic transplantation is associated with a high incidence of extramedullary relapses in multiple myeloma patients. Leukemia.

[34] Katodritou E., Dalampira D., Delimpasi S., Ntanasis-Stathopoulos I., Karaolidou F., Gkioka A.I., Labropoulou V., Spanoudakis E., Triantafyllou T., Kotsopoulou M. (2023). Update Analysis of Central Nervous System Multiple Myeloma Prognosis and Survival: A Real-World Multi-Institutional Study of the Greek Myeloma Study Group. Blood.

[35] Oshima K., Kanda Y., Nannya Y., Kaneko M., Hamaki T., Suguro M., Yamamoto R., Chizuka A., Matsuyama T., Takezako N. (2001). Clinical and pathologic findings in 52 consecutively autopsied cases with multiple myeloma. Am. J. Hematol..

[36] Liss B., Kutscher K. (2023). An unusual case of a multiple myeloma with thyroid involvement at primary diagnosis. Blood.

[37] You W.S., Bhuta S. (2021). Myeloma of Laryngeal Cartilage: Literature Review and Case Study. Ear Nose Throat J..

[38] Saidi I., El Idrissi Tourane L.o., Ait Batahar S., Amro L. (2022). A case of Multiple Myeloma with lung plasmacytoma. Respir. Med. Case Rep..

[39] Chim C.S., Wong W.M., Nicholls J., Chung L.P., Liang R. (2002). Extramedullary sites of involvement in hematologic malignancies: Case 3. Hemorrhagic gastric plasmacytoma as the primary presentation in multiple myeloma. J. Clin. Oncol..

[40] Abelman W., Virchis A., Yong K. (2005). Extramedullary myeloma representing as a pericardial effusion with tamponade: Two case reports and a further review of 19 cases in the literature. Leuk. Lymphoma.

[41] Wang X., Xie H., Zhang L. (2019). Multiple myeloma with onset of pancreas involvement: A case report. Medicine.

[42] Yamashita K., Horiuchi T., Hayashida A., Tachibana H., Toki D., Kondo T. (2019). Multiple myeloma with testicular involvement: A case report. Urol. Case Rep..

[43] Zhong Y.P., Zhang J.J., Huang X.N. (2012). Multiple myeloma with rupture of ovarian plasmacytoma. Chin. Med. J..

[44] Requena L., Kutzner H., Palmedo G., Calonje E., Requena C., Pérez G., Pastor M.A., Sangueza O.P. (2003). Cutaneous Involvement in Multiple Myeloma: A Clinicopathologic, Immunohistochemical, and Cytogenetic Study of 8 Cases. Arch. Dermatol..

[45] Aslaner Ak M., Erdemir R.U. (2022). Multiple muscle involvement in relapsed multiple myeloma: A rare case. J. Cancer Res. Ther..

[46] Mathew J., Lubitz S., Zaidan J. (2020). SUN-928 Adrenal Plasmacytoma in Multiple Myeloma Patient-An Unusual Presentation. J. Endocr. Soc..

[47] Zanwar S., Ho M., Lin Y., Kapoor P., Binder M., Buadi F.K., Dispenzieri A., Dingli D., Fonder A., Gertz M.A. (2023). Natural history, predictors of development of extramedullary disease, and treatment outcomes for patients with extramedullary multiple myeloma. Am. J. Hematol..

[48] Bergsagel P.L., Kuehl W.M. (2001). Chromosome translocations in multiple myeloma. Oncogene.

[49] Samur M.K., Aktas Samur A., Shah P., Park J., Fulciniti M., Shammas M.A., Corre J., Anderson K.C., Parmigiani G., Avet-Loiseau H. (2024). Development of hyperdiploidy starts at an early age and takes a decade to complete. Blood.

[50] Ho M., Patel A., Goh C.Y., Moscvin M., Zhang L., Bianchi G. (2020). Changing paradigms in diagnosis and treatment of monoclonal gammopathy of undetermined significance (MGUS) and smoldering multiple myeloma (SMM). Leukemia.

[51] Landgren O., Kyle R.A., Pfeiffer R.M., Katzmann J.A., Caporaso N.E., Hayes R.B., Dispenzieri A., Kumar S., Clark R.J., Baris D. (2009). Monoclonal gammopathy of undetermined significance (MGUS) consistently precedes multiple myeloma: A prospective study. Blood.

[52] Kyle R.A., Larson D.R., Therneau T.M., Dispenzieri A., Kumar S., Cerhan J.R., Rajkumar S.V. (2018). Long-Term Follow-up of Monoclonal Gammopathy of Undetermined Significance. N. Engl. J. Med..

[53] García-Ortiz A., Rodríguez-García Y., Encinas J., Maroto-Martín E., Castellano E., Teixidó J., Martínez-López J. (2021). The Role of Tumor Microenvironment in Multiple Myeloma Development and Progression. Cancers.

[54] Singh P., Mohammad K.S., Pelus L.M. (2020). CXCR4 expression in the bone marrow microenvironment is required for hematopoietic stem and progenitor cell maintenance and early hematopoietic regeneration after myeloablation. Stem Cells.

[55] Alsayed Y., Ngo H., Runnels J., Leleu X., Singha U.K., Pitsillides C.M., Spencer J.A., Kimlinger T., Ghobrial J.M., Jia X. (2007). Mechanisms of regulation of CXCR4/SDF-1 (CXCL12)-dependent migration and homing in multiple myeloma. Blood.

[56] Ghobrial I.M., Liu C.-J., Zavidij O., Azab A.K., Baz R., Laubach J.P., Mishima Y., Armand P., Munshi N.C., Basile F. (2019). Phase I/II trial of the CXCR4 inhibitor plerixafor in combination with bortezomib as a chemosensitization strategy in relapsed/refractory multiple myeloma. Am. J. Hematol..

[57] Aggarwal R., Ghobrial I.M., Roodman G.D. (2006). Chemokines in multiple myeloma. Exp. Hematol..

[58] Sahin A.O., Buitenhuis M. (2012). Molecular mechanisms underlying adhesion and migration of hematopoietic stem cells. Cell Adhes. Migr..

[59] Cerny J., Fadare O., Hutchinson L., Wang S.A. (2008). Clinicopathological features of extramedullary recurrence/relapse of multiple myeloma. Eur. J. Haematol..

[60] Chang H., Bartlett E.S., Patterson B., Chen C.I., Yi Q.L. (2005). The absence of CD56 on malignant plasma cells in the cerebrospinal fluid is the hallmark of multiple myeloma involving central nervous system. Br. J. Haematol..

[61] Dahl I.M., Rasmussen T., Kauric G., Husebekk A. (2002). Differential expression of CD56 and CD44 in the evolution of extramedullary myeloma. Br. J. Haematol..

[62] Sahara N., Takeshita A., Shigeno K., Fujisawa S., Takeshita K., Naito K., Ihara M., Ono T., Tamashima S., Nara K. (2002). Clinicopathological and prognostic characteristics of CD56-negative multiple myeloma. Br. J. Haematol..

[63] Corre J., Mahtouk K., Attal M., Gadelorge M., Huynh A., Fleury-Cappellesso S., Danho C., Laharrague P., Klein B., Reme T. (2007). Bone marrow mesenchymal stem cells are abnormal in multiple myeloma. Leukemia.

[64] Garayoa M., Garcia J.L., Santamaria C., Garcia-Gomez A., Blanco J.F., Pandiella A., Hernandez J.M., Sanchez-Guijo F.M., del Canizo M.C., Gutierrez N.C. (2009). Mesenchymal stem cells from multiple myeloma patients display distinct genomic profile as compared with those from normal donors. Leukemia.

[65] Binder M., Szalat R.E., Talluri S., Fulciniti M., Avet-Loiseau H., Parmigiani G., Samur M.K., Munshi N.C. (2024). Bone marrow stromal cells induce chromatin remodeling in multiple myeloma cells leading to transcriptional changes. Nat. Commun..

[66] Rajan A.M., Rajkumar S.V. (2015). Interpretation of cytogenetic results in multiple myeloma for clinical practice. Blood Cancer J..

[67] Vande Broek I., Vanderkerken K., Van Camp B., Van Riet I. (2008). Extravasation and homing mechanisms in multiple myeloma. Clin. Exp. Metastasis.

[68] Garcés J.J., San-Miguel J., Paiva B. (2022). Biological Characterization and Clinical Relevance of Circulating Tumor Cells: Opening the Pandora’s Box of Multiple Myeloma. Cancers.

[69] Bhutani M., Foureau D.M., Atrash S., Voorhees P.M., Usmani S.Z. (2020). Extramedullary multiple myeloma. Leukemia.

[70] Miyazaki K., Suzuki K. (2018). CD56 for Multiple Myeloma: Lack of CD56 May Be Associated with Worse Prognosis. Acta Haematol..

[71] McAvera R., Quinn J., Murphy P., Glavey S. (2023). Genetic Abnormalities in Extramedullary Multiple Myeloma. Int. J. Mol. Sci..

[72] Besse L., Sedlarikova L., Greslikova H., Kupska R., Almasi M., Penka M., Jelinek T., Pour L., Adam Z., Kuglik P. (2016). Cytogenetics in multiple myeloma patients progressing into extramedullary disease. Eur. J. Haematol..

[73] Shaughnessy J.D., Zhan F., Burington B.E., Huang Y., Colla S., Hanamura I., Stewart J.P., Kordsmeier B., Randolph C., Williams D.R. (2006). A validated gene expression model of high-risk multiple myeloma is defined by deregulated expression of genes mapping to chromosome 1. Blood.

[74] Sevcikova S., Paszekova H., Besse L., Sedlarikova L., Kubaczkova V., Almasi M., Pour L., Hajek R. (2015). Extramedullary relapse of multiple myeloma defined as the highest risk group based on deregulated gene expression data. Biomed. Pap. Med. Fac. Univ. Palacky. Olomouc Czech Repub..

[75] Billecke L., Murga Penas E.M., May A.M., Engelhardt M., Nagler A., Leiba M., Schiby G., Kroger N., Zustin J., Marx A. (2013). Cytogenetics of extramedullary manifestations in multiple myeloma. Br. J. Haematol..

[76] Chen T., Sun Z., Cui Y., Ji J., Li Y., Qu X. (2023). Identification of long noncoding RNA NEAT1 as a key gene involved in the extramedullary disease of multiple myeloma by bioinformatics analysis. Hematology.

[77] Egan J.B., Kortuem K.M., Kurdoglu A., Izatt T., Aldrich J., Reiman R., Phillips L., Baker A., Shi C.X., Schmidt J. (2013). Extramedullary myeloma whole genome sequencing reveals novel mutations in Cereblon, proteasome subunit G2 and the glucocorticoid receptor in multi drug resistant disease. Br. J. Haematol..

[78] Kriegova E., Fillerova R., Minarik J., Savara J., Manakova J., Petrackova A., Dihel M., Balcarkova J., Krhovska P., Pika T. (2021). Whole-genome optical mapping of bone-marrow myeloma cells reveals association of extramedullary multiple myeloma with chromosome 1 abnormalities. Sci. Rep..

[79] Liu Y., Jelloul F., Zhang Y., Bhavsar T., Ho C., Rao M., Lewis N.E., Cimera R., Baik J., Sigler A. (2020). Genetic Basis of Extramedullary Plasmablastic Transformation of Multiple Myeloma. Am. J. Surg. Pathol..

[80] Long X., Xu Q., Lou Y., Li C., Gu J., Cai H., Wang D., Xu J., Li T., Zhou X. (2020). The utility of non-invasive liquid biopsy for mutational analysis and minimal residual disease assessment in extramedullary multiple myeloma. Br. J. Haematol..

[81] Mithraprabhu S., Sirdesai S., Chen M., Khong T., Spencer A. (2018). Circulating Tumour DNA Analysis for Tumour Genome Characterisation and Monitoring Disease Burden in Extramedullary Multiple Myeloma. Int. J. Mol. Sci..

[82] Qu X., Chen L., Qiu H., Lu H., Wu H., Qiu H., Liu P., Guo R., Li J. (2015). Extramedullary manifestation in multiple myeloma bears high incidence of poor cytogenetic aberration and novel agents resistance. Biomed. Res. Int..

[83] Ryu D., Kim S.J., Hong Y., Jo A., Kim N., Kim H.J., Lee H.O., Kim K., Park W.Y. (2020). Alterations in the Transcriptional Programs of Myeloma Cells and the Microenvironment during Extramedullary Progression Affect Proliferation and Immune Evasion. Clin. Cancer Res..

[84] Smetana J., Oppelt J., Stork M., Pour L., Kuglik P. (2018). Chromothripsis 18 in multiple myeloma patient with rapid extramedullary relapse. Mol. Cytogenet..

[85] Sun Z., Ji J., Li Y., Cui Y., Fan L., Li J., Qu X. (2023). Identification of evolutionary mechanisms of myelomatous effusion by single-cell RNA sequencing. Blood Adv..

[86] Xia Y., Shi Y., Chen Z., Zhang J., Zhu Y., Guo R., Zhang R., Shi Q., Li J., Chen L. (2022). Characteristics and prognostic value of extramedullary chromosomal abnormalities in extramedullary myeloma. Chin. Med. J..

[87] Yao Q., Morgan G.J., Chim C.S. (2018). Distinct promoter methylation profile reveals spatial epigenetic heterogeneity in 2 myeloma patients with multifocal extramedullary relapses. Clin. Epigenetics.

[88] Jelinek T., Zihala D., Sevcikova T., Anilkumar Sithara A., Kapustova V., Sahinbegovic H., Venglar O., Muronova L., Broskevicova L., Nenarokov S. (2024). Beyond the marrow: Insights from comprehensive next-generation sequencing of extramedullary multiple myeloma tumors. Leukemia.

[89] Zanwar S., Novak J., Howe M.D., Binder M., Gonsalves W.I., Braggio E., Rajkumar V., Jevremovic D., Dasari S., Kumar S. (2024). The Mutational Landscape of Extramedullary Multiple Myeloma Reveals Novel Biologic Insights and Potential Therapeutic Targets for Exploration. Blood.

[90] Schavgoulidze A., Corre J., Samur M.K., Mazzotti C., Pavageau L., Perrot A., Cazaubiel T., Leleu X., Macro M., Belhadj K. (2024). RAS/RAF landscape in monoclonal plasma cell conditions. Blood.

[91] Paiva B., Calasanz M.-J. (2024). RASping myeloma genomics. Blood.

[92] Ansari-Pour N., Samur M., Flynt E., Gooding S., Towfic F., Stong N., Estevez M.O., Mavrommatis K., Walker B., Morgan G. (2023). Whole-genome analysis identifies novel drivers and high-risk double-hit events in relapsed/refractory myeloma. Blood.

[93] Maclachlan K.H., Garces J.-J., Shekarkhand T., Rajeeve S., Hashmi H., Hassoun H., Hultcrantz M., Korde N., Tan C.R., Mailankody S. (2024). Genomic Complexity Correlates with the Degree of Marrow Independence of Malignant Plasma Cells in the Context of Extramedullary Disease. Blood.

[94] Bianchi G., Czarnecki P.G., Ho M., Roccaro A.M., Sacco A., Kawano Y., Gullà A., Samur A.A., Chen T., Wen K. (2021). ROBO1 Promotes Homing, Dissemination, and Survival of Multiple Myeloma within the Bone Marrow Microenvironment. Blood Cancer Discov..

[95] Pinho A.V., Van Bulck M., Chantrill L., Arshi M., Sklyarova T., Herrmann D., Vennin C., Gallego-Ortega D., Mawson A., Giry-Laterriere M. (2018). ROBO2 is a stroma suppressor gene in the pancreas and acts via TGF-β signalling. Nat. Commun..

[96] He H., Di Y., Liang M., Yang F., Yao L., Hao S., Li J., Jiang Y., Jin C., Fu D. (2013). The microRNA-218 and ROBO-1 signaling axis correlates with the lymphatic metastasis of pancreatic cancer. Oncol. Rep..

[97] John M., Helal M., Duell J., Mattavelli G., Stanojkovska E., Afrin N., Leipold A.M., Steinhardt M.J., Zhou X., Žihala D. (2024). Spatial transcriptomics reveals profound subclonal heterogeneity and T-cell dysfunction in extramedullary myeloma. Blood.

[98] Qi Y., Li H., Qi K., Zhu F., Cheng H., Chen W., Yan Z., Li D., Sang W., Fei X. (2024). Clinical outcomes and microenvironment profiling in relapsed/refractory multiple myeloma patients with extramedullary disease receiving anti-BCMA CAR T-cell-based therapy. Am. J. Hematol..

[99] Kastritis E., Zervas K., Symeonidis A., Terpos E., Delimbassi S., Anagnostopoulos N., Michali E., Zomas A., Katodritou E., Gika D. (2009). Improved survival of patients with multiple myeloma after the introduction of novel agents and the applicability of the International Staging System (ISS): An analysis of the Greek Myeloma Study Group (GMSG). Leukemia.

[100] Kumar S.K., Rajkumar S.V., Dispenzieri A., Lacy M.Q., Hayman S.R., Buadi F.K., Zeldenrust S.R., Dingli D., Russell S.J., Lust J.A. (2008). Improved survival in multiple myeloma and the impact of novel therapies. Blood.

[101] Puertas B., González-Calle V., Sobejano-Fuertes E., Escalante F., Queizán J.A., Bárez A., Labrador J., Alonso-Alonso J.M., García de Coca A., Cantalapiedra A. (2023). Novel Agents as Main Drivers for Continued Improvement in Survival in Multiple Myeloma. Cancers.

[102] Yin J., Zhou X., Li X., Yuan C., Chu X., Hao L., Wu H., Zhong Y. (2024). Selinexor combined with bortezomib, lenalidomide, and dexamethasone for the treatment of newly diagnosed multiple myeloma with extramedullary disease. Sci. Rep..

[103] Munshi N.C., Anderson L.D., Shah N., Madduri D., Berdeja J., Lonial S., Raje N., Lin Y., Siegel D., Oriol A. (2021). Idecabtagene Vicleucel in Relapsed and Refractory Multiple Myeloma. N. Engl. J. Med..

[104] Berdeja J.G., Madduri D., Usmani S.Z., Jakubowiak A., Agha M., Cohen A.D., Stewart A.K., Hari P., Htut M., Lesokhin A. (2021). Ciltacabtagene autoleucel, a B-cell maturation antigen-directed chimeric antigen receptor T-cell therapy in patients with relapsed or refractory multiple myeloma (CARTITUDE-1): A phase 1b/2 open-label study. Lancet.

[105] Garfall A.L., Nooka A.K., van de Donk N.W.C.J., Moreau P., Bhutani M., Oriol A., Martin T.G., Rosiñol L., Mateos M.-V., Bahlis N. (2024). MM-336 Long-Term Follow-Up From the Phase 1/2 MajesTEC-1 Trial of Teclistamab in Patients With Relapsed/Refractory Multiple Myeloma (RRMM). Clin. Lymphoma Myeloma Leuk..

[106] Lesokhin A.M., Tomasson M.H., Arnulf B., Bahlis N.J., Miles Prince H., Niesvizky R., Rodrίguez-Otero P., Martinez-Lopez J., Koehne G., Touzeau C. (2023). Elranatamab in relapsed or refractory multiple myeloma: Phase 2 MagnetisMM-3 trial results. Nat. Med..

[107] Schinke C.D., Touzeau C., Minnema M.C., Donk N.W.C.J.v.d., Rodríguez-Otero P., Mateos M.-V., Rasche L., Ye J.C., Vishwamitra D., Ma X. (2023). Pivotal phase 2 MonumenTAL-1 results of talquetamab (tal), a GPRC5DxCD3 bispecific antibody (BsAb), for relapsed/refractory multiple myeloma (RRMM). J. Clin. Oncol..

[108] Pan D., Mouhieddine T.H., Fu W., Moshier E., Parekh S., Jagannath S., Rossi A.C., Richter J., Rodriguez C., Sanchez L.J. (2023). Outcomes after CAR T Cells in Multiple Myeloma Patients with Extramedullary and Paramedullary Disease. Blood.

[109] Dima D., Abdallah A.O., Davis J.A., Awada H., Goel U., Rashid A., DeJarnette S., Anwer F., Shune L., Raza S. (2024). Impact of Extraosseous Extramedullary Disease on Outcomes of Patients with Relapsed-Refractory Multiple Myeloma receiving Standard-of-Care Chimeric Antigen Receptor T-Cell Therapy. Blood Cancer J..

[110] Gagelmann N., Dima D., Merz M., Hashmi H., Ahmed N., Tovar N., Oliver-Caldés A., Stölzel F., Rathje K., Fischer L. (2024). Development and Validation of a Prediction Model of Outcome After B-Cell Maturation Antigen-Directed Chimeric Antigen Receptor T-Cell Therapy in Relapsed/Refractory Multiple Myeloma. J. Clin. Oncol..

[111] Nakashima J.Y., Khatri V., Cruz-Chamorro R.J., Zhou J., Patra P., Gonzalez R., De Avila G., Locke F.L., Liu H.D., Nishihori T. (2024). Patterns of Failure in Multiple Myeloma with Extramedullary Disease Following Anti-BCMA Directed Chimeric Antigen Receptor (CAR) T-Cell Therapy. Int. J. Radiat. Oncol. Biol. Phys..

[112] Vegivinti C.T.R., Lawrence Alexander Santhi J., Liu L., Keesari P.R., Thakur R., Hammami M.B., Kapu V., Pericherla S., Gopireddy M.M.r., Poojary N. (2023). Efficacy of Bispecific Antibodies Vs CAR-T in the Treatment of Extramedullary Disease and High-Risk Cytogenetics in Relapsed Multiple Myeloma: A Systematic Review and Meta-Analysis. Blood.

[113] Cohen A.D., Mateos M.V., Cohen Y.C., Rodriguez-Otero P., Paiva B., van de Donk N., Martin T., Suvannasankha A., De Braganca K.C., Corsale C. (2023). Efficacy and safety of cilta-cel in patients with progressive multiple myeloma after exposure to other BCMA-targeting agents. Blood.

[114] Raje N., Berdeja J., Lin Y., Siegel D., Jagannath S., Madduri D., Liedtke M., Rosenblatt J., Maus M.V., Turka A. (2019). Anti-BCMA CAR T-Cell Therapy bb2121 in Relapsed or Refractory Multiple Myeloma. N. Engl. J. Med..

[115] Gagelmann N., Ayuk F.A., Klyuchnikov E., Wolschke C., Berger S.C., Kroger N. (2023). Impact of high-risk disease on the efficacy of chimeric antigen receptor T-cell therapy for multiple myeloma: A meta-analysis of 723 patients. Haematologica.

[116] Li C., Xu J., Luo W., Liao D., Xie W., Wei Q., Zhang Y., Wang X., Wu Z., Kang Y. (2024). Bispecific CS1-BCMA CAR-T cells are clinically active in relapsed or refractory multiple myeloma. Leukemia.

[117] Wang Y., Cao J., Gu W., Shi M., Lan J., Yan Z., Jin L., Xia J., Ma S., Liu Y. (2022). Long-Term Follow-Up of Combination of B-Cell Maturation Antigen and CD19 Chimeric Antigen Receptor T Cells in Multiple Myeloma. J. Clin. Oncol..

[118] Zhang M., Zhou L., Zhao H., Zhang Y., Wei G., Hong R., Wu W., Xu H., Wang L., Ni F. (2021). Risk Factors Associated with Durable Progression-Free Survival in Patients with Relapsed or Refractory Multiple Myeloma Treated with Anti-BCMA CAR T-cell Therapy. Clin. Cancer Res..

[119] Que Y., Xu M., Xu Y., Almeida V.D.F., Zhu L., Wang Z., Wang Y., Liu X., Jiang L., Wang D. (2021). Anti-BCMA CAR-T Cell Therapy in Relapsed/Refractory Multiple Myeloma Patients With Extramedullary Disease: A Single Center Analysis of Two Clinical Trials. Front. Immunol..

[120] Li C., Cao W., Que Y., Wang Q., Xiao Y., Gu C., Wang D., Wang J., Jiang L., Xu H. (2021). A phase I study of anti-BCMA CAR T cell therapy in relapsed/refractory multiple myeloma and plasma cell leukemia. Clin. Transl. Med..

[121] Wang D., Wang J., Hu G., Wang W., Xiao Y., Cai H., Jiang L., Meng L., Yang Y., Zhou X. (2021). A phase 1 study of a novel fully human BCMA-targeting CAR (CT103A) in patients with relapsed/refractory multiple myeloma. Blood.

[122] Mei H., Li C., Jiang H., Zhao X., Huang Z., Jin D., Guo T., Kou H., Liu L., Tang L. (2021). A bispecific CAR-T cell therapy targeting BCMA and CD38 in relapsed or refractory multiple myeloma. J. Hematol. Oncol..

[123] Cohen A.D., Garfall A.L., Stadtmauer E.A., Melenhorst J.J., Lacey S.F., Lancaster E., Vogl D.T., Weiss B.M., Dengel K., Nelson A. (2019). B cell maturation antigen-specific CAR T cells are clinically active in multiple myeloma. J. Clin. Investig..

[124] Xu J., Chen L.J., Yang S.S., Sun Y., Wu W., Liu Y.F., Xu J., Zhuang Y., Zhang W., Weng X.Q. (2019). Exploratory trial of a biepitopic CAR T-targeting B cell maturation antigen in relapsed/refractory multiple myeloma. Proc. Natl. Acad. Sci. USA.

[125] Brudno J.N., Maric I., Hartman S.D., Rose J.J., Wang M., Lam N., Stetler-Stevenson M., Salem D., Yuan C., Pavletic S. (2018). T Cells Genetically Modified to Express an Anti-B-Cell Maturation Antigen Chimeric Antigen Receptor Cause Remissions of Poor-Prognosis Relapsed Multiple Myeloma. J. Clin. Oncol..

[126] Riedhammer C., Bassermann F., Besemer B., Bewarder M., Brunner F., Carpinteiro A., Einsele H., Faltin J., Frenking J., Gezer D. (2024). Real-world analysis of teclistamab in 123 RRMM patients from Germany. Leukemia.

[127] Mohan M., Monge J., Shah N., Luan D., Forsberg M., Bhatlapenumarthi V., Balev M., Patwari A., Cheruvalath H., Bhutani D. (2024). Teclistamab in relapsed refractory multiple myeloma: Multi-institutional real-world study. Blood Cancer J..

[128] Joiner L., Bal S., Godby K.N., Costa L.J. (2023). Teclistamab in patients with multiple myeloma and impaired renal function. Am. J. Hematol..

[129] Moreau P., Garfall A.L., van de Donk N., Nahi H., San-Miguel J.F., Oriol A., Nooka A.K., Martin T., Rosinol L., Chari A. (2022). Teclistamab in Relapsed or Refractory Multiple Myeloma. N. Engl. J. Med..

[130] Touzeau C., Krishnan A.Y., Moreau P., Perrot A., Usmani S.Z., Manier S., Cavo M., Martinez Chamorro C., Nooka A.K., Martin T.G. (2024). Efficacy and safety of teclistamab in patients with relapsed/refractory multiple myeloma after BCMA-targeting therapies. Blood.

[131] Sidana S., Patel K.K., Peres L.C., Bansal R., Kocoglu M.H., Shune L., Atrash S., Smith K., Midha S., Ferreri C. (2025). Safety and efficacy of standard-of-care ciltacabtagene autoleucel for relapsed/refractory multiple myeloma. Blood.

[132] Richard S., Lancman G., Rossi A., Chari A., Parekh S., Sanchez L., Rodriguez C., Cho H.J., Richter J., Thibaud S. (2022). Extramedullary Relapse Post CAR-T. Blood.

[133] Costa L.J., Bahlis N.J., Usmani S.Z., van de Donk N.W.C.J., Nooka A.K., Perrot A., Qi K., Hodin C., Uhlar C., Zuppa A. (2024). MM-328 Efficacy and Safety of Teclistamab in Patients With Relapsed/Refractory Multiple Myeloma (RRMM) With High-Risk (HR) Features: A Subgroup Analysis From the Phase 1/2 MajesTEC-1 Study. Clin. Lymphoma Myeloma Leuk..

[134] Dima D., Davis J.A., Ahmed N., Jia X., Sannareddy A., Shaikh H., Shune L., Kaur G., Khouri J., Afrough A. (2024). Safety and Efficacy of Teclistamab in Patients with Relapsed/Refractory Multiple Myeloma: A Real-World Experience. Transplant. Cell Ther..

[135] Cohen Y.C., Magen H., Gatt M., Sebag M., Kim K., Min C.-K., Ocio E.M., Yoon S.-S., Chu M.P., Rodríguez-Otero P. (2025). Talquetamab plus Teclistamab in Relapsed or Refractory Multiple Myeloma. N. Engl. J. Med..

[136] Mejia Saldarriaga M., Jayabalan D.S., Sowa A., Monge J., Rosenbaum C.A., Pearse R.N., Niesvizky R., Patel S., Bustoros M. (2023). Genomic landscape of multiple myeloma with extramedullary disease: Results from a large patient database. J. Clin. Oncol..

[137] van de Donk N.W.C.J., Minnema M.C., van der Holt B., Schjesvold F., Wu K.L., Broijl A., Roeloffzen W.W.H., Gadisseur A., Pietrantuono G., Pour L. (2023). Treatment of primary plasma cell leukaemia with carfilzomib and lenalidomide-based therapy (EMN12/HOVON-129): Final analysis of a non-randomised, multicentre, phase 2 study. Lancet Oncol..

[138] Giesen N., Chatterjee M., Scheid C., Poos A.M., Besemer B., Miah K., Benner A., Becker N., Moehler T., Metzler I. (2023). A phase 2 clinical trial of combined BRAF/MEK inhibition for BRAFV600E-mutated multiple myeloma. Blood.

[139] Heuck C.J., Jethava Y., Khan R., van Rhee F., Zangari M., Chavan S., Robbins K., Miller S.E., Matin A., Mohan M. (2016). Inhibiting MEK in MAPK pathway-activated myeloma. Leukemia.

[140] Costa L.J., Schjesvold F., Popat R., Siegel D., Usmani S.Z., Ali S.A., Chu M.P., Hartley-Brown M.A., Bahlis N.J., Oriol A. (2024). Mezigdomide (MEZI) in Novel-Novel Combinations for Relapsed or Refractory Multiple Myeloma (RRMM): Preliminary Results from the CA057-003 Trial. Blood.

